# Luminescent and fluorescent triple reporter plasmid constructs for Wnt, Hedgehog and Notch pathway

**DOI:** 10.1371/journal.pone.0226570

**Published:** 2019-12-20

**Authors:** Julia Maier, Salma Elmenofi, Alexander Taschauer, Martina Anton, Haider Sami, Manfred Ogris

**Affiliations:** 1 Laboratory of MacroMolecular Cancer Therapeutics (MMCT), Center of Pharmaceutical Sciences, Department of Pharmaceutical Chemistry, University of Vienna, Althanstrasse, Vienna, Austria; 2 Institutes of Molecular Immunology and Experimental Oncology, Klinikum rechts der Isar, Technische Universität München, Munich, Germany; National Cancer Center, JAPAN

## Abstract

Tracking the activity of signalling pathways is a fundamental method for basic science, as well as in cancer- and pharmaceutical research. The developmental pathways Wnt, Hedgehog and Notch are frequently deregulated in cancers and represent a valuable target for the discovery of novel anticancer compounds. Here we present reporter systems for tracking activity of these pathways by using specific promoter elements driving the expression of either sensitive luciferases or fluorescent proteins. A high level of sensitivity was obtained using the luciferase reporter genes for firefly (FLuc), secreted *Gaussia* (GLuc) and synthetic NanoLuc (NLuc). As fluorescent reporter proteins, mTurqouise2, tdTomato and iRFP720 were chosen. Specificity of pathway activity was validated by co-transfection with pathway activating genes, showing significant response to induction. In addition, multi-gene plasmids were cloned, allowing the detection of all three pathways by one vector. By using the multi-gene vector 3P-Luc (wnt-NLuc, hedgehog-FLuc, Notch-GLuc), we could unambiguously demonstrate the crosstalk between pathways, while excluding cross reactivity of luciferase substrates. First studies with synthetic compounds confirmed the applicability of the system for future drug screening approaches.

## Introduction

Dysregulation of developmental pathways plays a pivotal role in cancer [1]. Up-regulation of Wnt, for example, correlates with dismal prognosis in a broad range of solid cancers, leading to development of several Wnt inhibitors and their evaluation within advanced clinical studies [2]. More broadly, targeting the Wnt, Hedgehog (Hh) and Notch pathway has been described as a successful approach in cancer therapy [3]. It is speculated that such an approach might also be effective against therapy-resistant cancer cell sub-populations, such as cancer initiating cells, which are known to show deregulation of these three pathways [4]. In the search of novel treatment options, compound screening libraries are nowadays often employed. These allow the selection of suitable molecular structures from millions of compounds using appropriate *in silico* models [5]. Nevertheless, initial virtual screening results need to be validated and further screened in biological systems. For this purpose, reporter genes expressed under the control of pathway specific response elements are often employed. Regulation of pathways mostly depends on the activity of transcription factors binding to promoter elements. Hence, several natively occurring [6], but also synthetic promoter elements have been designed to drive expression of reporter genes [7]. To give an example, the canonical Wnt pathway is activated after binding of Wnt signal proteins (e.g. Wnt3a) to its receptor frizzled and the LPR co-receptors [8]. Factors downstream of LPR inactivate the β-catenin destruction complex. The stabilised β-catenin accumulates and translocates to the nucleus, where it forms an active complex with LEF (lymphoid enhancer factor) and TCF (T-cell factor) leading to a transcriptional switch. Hence, the most commonly used Wnt reporter is a synthetic promoter element with multiple TCF/LEF binding sites [9]. Similarly, the Sonic Hedgehog pathway is initiated by binding of SHH protein to its receptor Patched (PTCH), which releases the PTCH inhibition of Smoothened (Smo). Smo in turn activates Gli transcription factors, which after promoter binding initiate expression of hedgehog target genes. Here, the most prominent reporter is based on multiple GLI binding sites [10]. In case of Notch pathway, after ligand binding the Notch receptor sheds its intracellular domain (NICD), which translocates to the nucleus and associates with DNA binding proteins like CBF and activates Notch related gene expression. Hence, CBF promoters can be used for Notch reporter expression purposes [11].

For reporter systems, all of the aforementioned promoter elements have been mostly used in conjunction with firefly luciferase, which usually requires the lysis of cells or analysis of the supernatant. With luciferases, a very high signal to noise ratio is obtained, and they can be utilised both in cell culture, but also *in vivo* [12]. On the other hand, fluorescent reporter proteins covering the whole range from UV, visible light and near infrared [13] enable activity measurements in individual cells by flow cytometry or, using fluorescence microscopy, also in tissues, but with a considerably lower signal to noise ratio when compared with luciferase assays. For many applications in developmental- and cancer biology, multi-reporter gene constructs would be desirable, allowing the simultaneous analysis of interconnected pathways. Also for Wnt, Hh and Notch it is supposed that their interplay is crucial, especially in therapy-resistant subsets of cancer cells, and drug discovery is ongoing [3]. With the technical advances in developing diode pumped lasers, several easy to use flow cytometers have been developed being equipped with three or more laser allowing multiplex analysis [14]. The excitation wavelengths usually range from near UV (405 nm, violet laser), blue (488 nm) to dark red (638 nm), allowing simultaneous detection of several distinct fluorescent proteins [15]. For luciferases, multiplexing is possible when combining enzymes requiring different substrates. An established approach is normalization of firefly luciferase activity (FLuc, substrate luciferin) by co-transfection with *Renilla* luciferase (*RLuc*, substrate colenterazine) [12]. Signal separation is achieved harnessing the differences in enzyme kinetics, where *RLuc* exhibits a flash kinetic, and FLuc a glow type kinetic. An alternative coelenterazine driven, ATP independent luciferase is derived from *Gaussia princeps* (GLuc), offering a significantly higher signal [16]. GLuc is secreted in its native form, allowing also physical separation form cytoplasmic FLuc. More recently, NanoLuc (NLuc), derived from *Oplophorus gracilirostris*, has been introduced [17]. This extremely bright, ATP independent luciferase with glow type luminescence requires the imidazopyrazinone based substrate furimazine. Hence, luciferase reporter can be combined differing in substrate requirements, cellular trafficking (secreted vs cytoplasmic) and enzyme kinetics [18]. During embryonic development, but also in cancer stem cells, there is an intense crosstalk taking place between Wnt, Notch and Hedgehog [3]. Being able to simultaneously track all of them offers the opportunity to tune drug regimens in a way to allow either specific interference with one, or simultaneously with all pathways. Towards this goal of tracking multiple pathways at the same time, transient and stable co-transfection with several luciferase reporter based plasmids is feasible, however position effects occur when expression cassettes integrate at different genomic sites [19]. To avoid such effects and allow a direct comparison of different promoter elements, use of insulator elements is one option. Alternatively, plasmids with multiple expression cassettes can be generated with new cloning techniques, such as the MultiSite Gateway cloning technology, simplifying the development of such complex multi-gene vectors [20]. However, it is critical to compare the performance of reporters within multiple expression cassettes to their single-expression counterparts, so as to assess their applicability. Here, we present the development of single and multi-gene vector constructs for Wnt, Hh and Notch reporter constructs with either luciferases or fluorophores as reporter genes. Their specificity and performance in transfection assays was evaluated and several compounds known to interfere with the distinct pathway tested.

## Material and methods

### Cells

HeLa (#CCL2, ATCC, Manassas, US) and 293T (#CRL-3216, ATCC) cells were cultured in DMEM/4.5g/L glucose (#D5671, Sigma-Aldrich, Darmstadt, Germany) with 10% heat inactivated fetal bovine serum (FBS, #S181H, Biowest, Nuaillé, France), 2% L-Glutamin (#G7513, Sigma-Aldrich) and 1% Penicillin/Streptomycin (#P0781, Sigma-Aldrich) at 37°C, 5% CO_2_ and saturated humidity. L Wnt-3A cells (#CRL-2647, ATCC) were maintained under the same conditions, with 0.4mg/ml G-418 (#G418-RO, Sigma Aldrich) supplemented in the medium. Conditioned medium (CM) for activation of the Wnt pathway was produced as described [21]. In brief, confluent Wnt-3A cells were incubated with culture medium containing G-418 consecutively for four and three days. Both media batches were sterile filtered (0.2 μm), mixed with equal volumes and stored frozen until further use.

### Plasmids

The 12GLI-RETKO-luc reporter was a gift from Peter Zaphiropoulos (GLI-RET) [22], CBF:H2B-Venus from Anna-Katerina Hadjantonakis (CBF-Ven, Addgene plasmid #44211) [23], CBFRE (mt) EGFP from Nicholas Gaiano (Cmu-eG, Addgene plasmid #26870) [24], EF.hICN1.CMV.GFP from Linzhao Cheng (Addgene plasmid #17623) [25], hGli1 flag3x from Martin Fernandez-Zapico (phGli1, Addgene plasmid #84922), human beta-catenin pcDNA3 from Eric Fearon (pβ-cat, Addgene plasmid #16828) [26], M50 Super 8x TOPFlash (TOPFlash, Addgene plasmid #12456) and M51 Super 8x FOPFlash (FOPFlash, Addgene plasmid #12457) were gifts from Randall Moon [27]. pAd-Wnt3a was a gift from Tong-Chuan He (pWnt3a, Addgene plasmid #12518) [28] and pCAGGS-NICD from Nicholas Gaiano (pNICD, Addgene plasmid #26891) [29]. pCMV-Gluc, a secreted variant, was acquired from New England Biolabs (NEB, # N8081S, Frankfurt am Main, Germany). The plasmid pEGFPLuc was acquired from Clontech (Mountain View, USA). pGL3b hPtch1 prom wt (PT wt) and pGL3-PTCH (PTCH1_VAR, mutated GLI binding sites, PT mut) were gifts from Fritz Aberger [30]. pLUT7 HA-Gli1 was a gift from Michael Ruppert (pHA-Gli1, Addgene plasmid #62970) [10], pLV-beta-catenin deltaN90 from Bob Weinberg (pβ-catΔ90, Addgene plasmid #36985) [31], pmTurquoise2-NES from Dorus Gadella (Addgene plasmid#36206) [32]. Promoterless pNL1.1 (#N1001) was obtained from Promega (Mannheim, Germany). pUC19 was included in the One Shot TOP10 set (#C404003) from Thermo Fisher Scientific (Schwerte, Germany). All remaining plasmids are content of the Multiple Lentiviral Expression System Kit, which was a gift from Ian Frew (Addgene kit #1000000060) [20]. In silico cloning and plasmid map generation was performed using SnapGene (v3.1.4, GSL Biotech LLC, Chicago, USA).

### Cloning

Primers were synthesized by Microsynth (Microsynth Austria GmbH, Vienna, Austria). All restriction enzymes were fast-digest variants from ThermoFisher Scientific. For preparative PCR, Q5 high-fidelity DNA polymerase (NEB, #M0492S) was used. Digests and PCR products were routinely gel purified in 0.7%-1.5% agarose gels (#443666A, VWR, Vienna, Austria) in sodium borate buffer at 80V. For gel extractions, a commercial kit (#K0692, ThermoFisher Scientific) was used as per the manual. Ligations were performed with T4 DNA ligase from ThermoFisher Scientific (#EL0011) or Blunt/TA ligase from NEB (#M0367S). For LR reactions, LR clonase plus from ThermoFisher Scientific (#12538120) was used as per the manual. Plasmids from LR reactions were transformed into chemically competent *E*.*coli* One Shot Mach1 (#C862003, ThermoFisher Scientific) as per manufacturer’s instructions. All other plasmids were propagated either in *E*.*coli* 10-beta (#C3019I, NEB) or DH5α after heat shock transformation. All bacteria were expanded in LB medium with appropriate antibiotics, and plasmids isolated with commercial miniprep or maxiprep kits (ThermoFisher Scientific, #K0503 and #K0492). All plasmids were characterized and validated by analytical restriction digests, and additional sequencing (Microsynth), if required.

### Luciferase reporter cloning

pMuLE_ENTR_TOP-NLuc1.1_L5-L4 (TOP-NLuc, for Wnt pathway): pMuLE_ENTR_MCS_L5-L4 was digested with KpnI and BamHI (pMuLE backbone, 2600 bp), M50 Super 8x TOPFlash with KpnI and Hind III (TOP promoter, 214bp), and pNL1.1 with HindIII and BamHI (NLuc cDNA, 822 bp). The three target fragments were ligated together to yield the final plasmid.

pMuLE_ENTR_12GLI-FLuc_R4-R3 (GLI-FLuc, for Hh pathway): A PCR of 12GLI-RETKO-luc was performed (forward primer: gaggagctctatacactccgctatcgc, reverse primer: tacactagtcagcagatgaacactgac). The resulting fragment was cut by SacI and SpeI (12GLI-Fluc expression cassette, 4000 bp), and cloned into a SacI/SpeI digested pMuLE_ENTR_MCS_R4-R3 (pMuLE backbone, 2800 bp).

pMuLE_ENTR_CBF-GLuc_L3-L2 (CBF-GLuc, for Notch pathway): pMuLE_ENTR_MCS_L3-L2 was cut with EcoRI and XhoI (pMuLe backbone, 2600 bp). A segment of CBF:H2B-Venus was PCR amplified (forward primer: aatgaattcgtattaccgccatgc, reverse primer: ccttagtcaccgccttct) and digested with EcoRI and HindIII (CBF promoter, 416 bp). pCMV-Gluc was cut with HindIII and XhoI (GLuc cDNA, 596 bp). The three pieces were ligated to yield the final plasmid.

pMuLE_EXPR_CMV-eGFP_TOP-NLuc1.1_GLI-FLuc_CBF-GLuc (3 pathway-luciferases, 3P-Luc, for Wnt, Hh and Notch pathways): An LR reaction of pMuLE_Lenti_Dest_Neo, pMuLE_ENTR_CMV-eGFP_L1-R5, and the three abovementioned ENTR luciferase reporter plasmids was performed.

### Fluorophore reporter cloning

pMuLE_ENTR_TOP-iRFP_L5-L4 (TOP-iRFP, for Wnt pathway): pMuLE_ENTR_MCS_L5-L4 was cut with KpnI and SpeI (pMuLE backbone, 2600 bp). A PCR of M50 Super 8x TOPFlash was performed (forward primer: cgcacgcactaggtaccgagctcttacgc, reverse primer: gcccagctgatggtggctttaccaacagt) and the fragment digested with KpnI and PvuII (TOP promoter, 253 bp). A segment of pMuLE_ENTR_SV40-iRFP_L3-L2 was also PCR amplified (forward primer: ataacagctgcaccatggcggaaggat, reverse primer: tggaactagtgactcactcttccatcacgc) and digested with PvuII and SpeI (iRFP cDNA, 962 bp). The three fragments were then ligated to yield the final plasmid.

pMuLE_ENTR_PTCH1-mTurquoise2_R4-R3 (PT-mT2, for Hh pathway): A segment of pmTurquoise2-NES was PCR amplified (forward primer: tatgaagcttatggtgagcaagggcg; reverse primer: gtatggatcctctacaaatgtggtatggctga) and digested with HindIII and BamHI (mTurquoise2 cDNA, 817 bp). pGL3b hPtch1 prom wt was digested with KpnI and HindIII (pPtch1 promoter, 1400 bp). In a first ligation step, the Ptch1 and mTurquoise2 fragments were ligated and then PCR amplified (forward primer: taaggtaccgcgtgctagagcttgcat; reverse primer: gtatggatcctctacaaatgtggtatggctga), The amplified fragment was subsequently digested with KpnI and BamHI (Ptch1-mTurquoise2, 2200 bp) and cloned into KpnI/BamHI digested pMuLE_MCS_R4-R3 (backbone, 2800 bp).

pMuLE_ENTR_CBF-tdTomato_L3-L2 (CBF-tdT, for Notch pathway): pMuLE_ENTR_SV40_tdtomato_L3-L2 was PCR amplified (forward primer: gcctctgagctattccagaagta; reverse primer: gagcatatggacacacattccacagcaac) to introduce a new NdeI site, and digested with BamHI/NdeI (backbone plus tdTomato, 4100 bp fragment). Similarly, a fragment of CBF:H2B-Venus was obtained by PCR (forward primer: tattggatcctggctctggcatgaattc; reverse primer: gtgacatatgataaccgtattaccgccatg), digested with the same enzymes (CBF promoter, 446 bp), and cloned into the backbone.

pMuLE_EXPR_CMV-eGFP_TOP-iRFP_PTCH1-mT2_CBF-tdT (3 pathway fluorophores, 3P-Fluor, for Wnt, Hh and Notch pathways): pMuLE_Lenti_Dest_Neo, pMuLE_ENTR_CMV-eGFP_L1-R5, and the three abovementioned ENTR fluorophore reporter plasmids were combined in an LR reaction.

### Auxilliary plasmids

EF.hICN1 (phICN1): For easy usage in flow cytometry, the CMV.GFP of the plasmid EF.hICN1.CMV.GFP was removed by digestion with EcoRI (9.2kb fragment), after which it was ligated. pUC19 and pDest were used as control or stuffer plasmids in transfections.

### 3P plasmid system

A set of plasmids generated in this work, including maps and sequences, can be found at Addgene.org (see data including Addgene plasmid numbers in S1 Table), while maps and the *in silico* generated sequence for CBF-GLuc (S1A Fig, S1 File) and 3P-Luc (S1B Fig, S2 File) are also in the supplementary information.

### Transfections

If not mentioned otherwise, 293T cells were used. Cells were seeded in white 96-well plates (Greiner Bio-One, #655098) for luciferase reporter assays or in transparent well plates (Greiner Bio-One, #655160) for fluorophore reporter assays. Twenty-four hours after seeding, cells were transfected using linear polyethylenimine (LPEI) in principle as described [33]. Growth media was exchanged for 100 μl serum-free DMEM high glucose. Polyplexes were generated after mixing all relevant plasmids at a final plasmid concentration of at least 20 μg/ml in HBS buffer at N/P 9, and further diluted with HBS after particle formation when required. If not described otherwise, 100 μl DMEM high glucose with 1% FBS and 2% L-Glutamine were added four hours after transfection, and cells further incubated. If not stated otherwise, the evaluation of the pathways was performed 24h after transfection for the Wnt pathway, and 48h after transfection for Hh and Notch pathways.

### Luciferase reporter assays

All plasmids used for luciferase transfection are summarised as supplementary material (S2 Table). For single pathway reporters, transfections were carried out as follows: For transfections with readout for FLuc (luciferin) or NLuc (furimazine), cells were transfected with polyplexes containing 50 ng reporter plasmid (encoding for FLuc or NLuc), 10 ng pCMV-GLuc (GLuc activity as normalizer) and 25 ng pUC19 or the indicated inducer plasmid. For transfections with readout for GLuc (coelenterazine), cells were transfected with polyplexes containing 50 ng reporter plasmid (encoding for GLuc), 50 ng pEGFPLuc (FLuc activity as normalizer) and 25 ng pUC19 or the indicated inducer plasmid. Care was taken not to detach cells when aspirating liquid, as this can negatively influence the signal normalization. All luminescence measurements were performed with an infinite M200Pro plate reader (Tecan, Grödig, Austria).

For GLuc activity measurements, 20 μl supernatant was transferred to a new white 96-well plate, 50 μl coelenterazine buffer (20μM coelenterazine in PBS supplemented with 5mM NaCl, pH 7.2 as per Tannous et al, ref [34]) added per well and the signal acquired for 10 seconds after a wait time of 2 seconds. For NLuc measurements, 160 μl medium were removed leaving 40 μl in the well. Then, 40 μl of Nano-Glo reagent (Promega, #N1110) was added, incubated for 3 min at RT and signal acquired for 10 seconds. For FLuc measurements, supernatant was completely aspirated. Cells were lysed in 30 μl 1x Passive Lysis Buffer (Promega, #E194A) and shaken for 30min at 500RPM at RT on an Eppendorf ThermoMixer C. For the measurement, 100 μl of luciferase assay reagent [35] were injected and the signal acquired for 10 seconds after a lag time of 2 seconds.

For the 3P-Luc based experiments and their comparison with individual plasmids, cells were co-transfected with 50 ng reporter plasmid plus 25 ng of respective inducer plasmid or pUC19. Luciferase activities were measured as described above. All luciferase activities were normalised to total cell viability using the CellTiter Fluor kit (Promega #G6081). For this, supernatant was completely aspirated and, if required, GLuc measured as described above. 100 μl of PBS and 20 μl 5x CellTiter-Fluor reagent was added, and plates incubated up to 2.5h at cell culture conditions. The complete supernatant was then transferred to a black 96-well plate (Greiner Bio-One, #655209) and fluorescence measured as by manufacturers recommendation. Afterwards, the cells were utilised for FLuc or NLuc measurements.

Signal normalization was carried out as follows: First, from each reporter and normalizer value, background was subtracted. Background values were obtained from untransfected cells measured with the indicated assay (furimazine, luciferin of coelenterazine based). An average background value was then substracted from each measured value. Per well, RLUs from reporter were divided by RLUs (or fluorescence units) from the normalisation method. For better graphical representation, the result was multiplied to give values of similar magnitude, using the same factor within one parameter comparison. For single reporter experiments, CMV-driven luciferases were used to normalize RLU values per well.

### Fluorophore reporter assays

All plasmids used for fluorophore transfection are summarised as supplementary material (S2 Table). 400ng of the reporter plasmid and 400ng pUC19 or the respective inducer were co-transfected per well. For the measurement, cells were washed once with PBS, detached with Versene (#15040–33, ThermoFisher Scientific), and the cell suspension transferred into a PCR plate (Nerbe, #04-083-0150, nerbe plus GmbH, Winsen, Germany). Cells were measured on a Macs-Quant Analyzer 10 flow cytometer (Miltenyi BiotecGmbH, Bergisch Gladbach, Germany) equipped with solid state laser emitting at 488 nm (FSC, SCC, B1-B3), 405 nm (V1 and V2) and 638 nm (R1 and R2). Plates were actively cooled for the whole measurement to 4°C using an Inheco CPAC cooling unit (INHECO, Planegg, Germany). Analysis of flow cytometry data was done with FlowJo 10.1r5 (FlowJo LLC, Ashland OR, USA). All signals were acquired in area (A) and height (H) mode. As a gating strategy, the main cell population was gated first in FSC-A versus (vs.) SSC-A, and single cells were further selected in FSC-A vs. FSC-H. mTurquoise2 was gated in V1-A vs. V2-A (V1: 450/50 nm bandpass (BP), V2: 525/50nm BP), tdTomato in B1-A (525/50 nm BP) vs. B3-A (655–730 nm BP), and iRFP in R1-A (655–730 nm BP) vs R2-A (750nm logpass (LP)). For analysis of 3P-Fluor, compensation matrices were generated in FlowJo by separately analyzing CMV-driven variants of all four colours. The appropriate matrix was then applied to the measured values of 3P-Fluor before analysis.

### Small molecules evaluation

The following small molecules were evaluated for induction or reduction of pathway activity. Wnt: CHIR99021(#SML1046) [36], LY2090314 (#SML1438) [36], niclosamide (N3510) [37], LGK-974 (#S7143) [36]; Hh: SAG dihydrochloride (SAG, #SML1314) [38], sonidegib (#S2151) [39], vismodegib (#S1082) [40]; Notch: suberohydroxamic acid (SBHA, #390585) [41], DAPT (#S2215) [42], Dibenzazepine (DBZ, #S2711) [43], valproic acid sodium salt (VPA, #P4543) [44]. All reagents were purchased from Selleckchem (Munich, Germany) or Sigma Aldrich. VPA and SAG were dissolved in ultrapure water (Arium pro, Sartorius AG, Göttingen, Germany), and the remaining compounds in DMSO (#D5879, Sigma-Aldrich).

Transfections and evaluations were performed as described above, with the following changes: For induction of the Wnt-pathway in the respective wells, 100 μl conditioned medium (containing wnt3a, see above) was added to the wells four hours after transfection. Compounds were also added four hours after transfection, at a final concentration of 10μM. An equal amount of appropriate solvent (water or DMSO) was added to the solvent control conditions.

### Statistical analysis

If not mentioned otherwise, all experiments were at least performed twice, with each condition at least in triplicate. Statistical analysis was performed using GraphPad Prism, version 7.02 (GraphPad Software La Jolla, USA) or an on-line tool (https://www.socscistatistics.com/). For fold induction analysis, results from identical experiments were pooled. Statistical analysis consisted of an unpaired t-test, columns show mean values, and error bars denominate standard deviation.

## Results

### Performance of single luciferase pathway reporters

**Fig 1:**
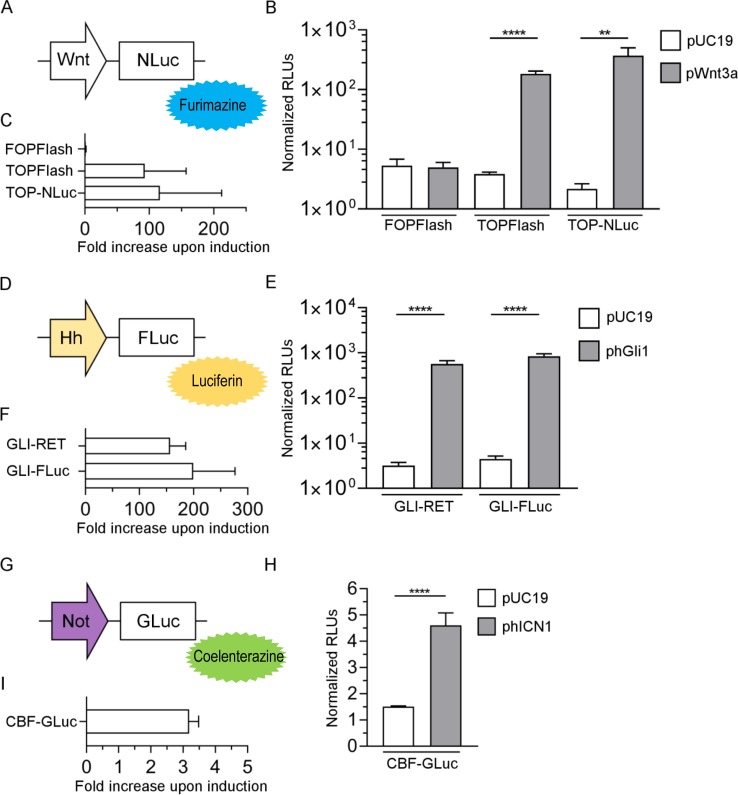
Evaluation of Wnt, Hh and Notch luciferase single reporters via luminescence readout. (A) The Wnt promoter TOP was combined with NLuc (substrate: furimazine). (D) The Hh promoter GLI-RET was backbone switched to obtain GLI-FLuc (substrate: luciferin). (G) For the Notch (Not) reporter construct, the CBF promoter was combined with GLuc (substrate: coelenterazine). All transfections were carried out in 293T cells. B, E, H: Luminescence measurement of cells transfected with the indicated plasmid and co-transfected with either pUC19 control or the indicated inducer plasmid. C, F, I: fold increase in signal for the indicated plasmid upon co-transfection with pUC19 control versus inducer plasmid (C: pWnt3a; F: phGli1; I: phICN1). In B, E and H results are show from a representative experiment; C, F and I are the average of 2–5 independent experiments. (**P≤0.01, ****P≤0.0001, ns = not significant, t-test, n = 4). Normalised RLUs were multiplied with 10 (CBF-GLuc), 100 (TOP-NLuc) or 1000 (FOPFlash, TOPFlash, GLI-RET and GLI-FLuc).

The Wnt-pathway sensitive TOP promoter was used to drive NLuc expression (Fig 1). Several plasmids were first evaluated regarding their ability to induce the Wnt pathway, with pWnt3a performing best (S2A Fig) and was thus selected for further experiments. The sensitivity of TOP-NLuc to Wnt-pathway induction (by co-transfection with pWnt3a) was compared with the established Wnt reporter TOPFlash (Fig 1B and 1C). The normalized RLUs of TOPFlash and TOP-NLuc were comparable in side-by side experiments with similar signal induction of approximately 100-fold (Fig 1B). The control plasmid FOPFlash, having mutant TCF/LEF binding sites, did not show a significant signal increase upon induction, as expected.

As luminescence-based Hh reporter, GLI-FLuc was generated (Fig 1D). For induction, several plasmid-based inducers were evaluated (pUC19 and pDest were used as control plasmids), with phGli1 offering the most consistent results (S2B Fig). The original reporter GLI-RET and the new GLI-FLuc showed similar performance, both in terms of expression strength (Fig 1E) and fold increase in signal after induction with phGli1 (Fig 1F).

The plasmid CBF-GLuc was cloned for tracking Notch activity (Fig 1G). Two different Notch-inducing plasmids, phICN1 and pNICD, were evaluated (S2C Fig) and phICN1 was chosen for further experiments. Although increase in normalized RLUs and fold increase upon induction of CBF-GLuc were considerably lower compared to TOP-NLuc and GLI-FLuc, results were statistically significant, and consistent over several experiments (Fig 1H and 1I). In contrast to the plasmids described above, a direct comparison of CBF-GLuc with CBF-based luciferase reporter was not feasible, as the original version is a fluorophore-based reporter.

Taken together, the newly developed pathway-sensitive luciferase based reporters perform equally well when compared to their original counterparts where applicable, and all show statistically significant responsiveness as well as robust fold-increase when induced with appropriate pathway activators.

### Performance of single fluorophore pathway reporters

**Fig 2:**
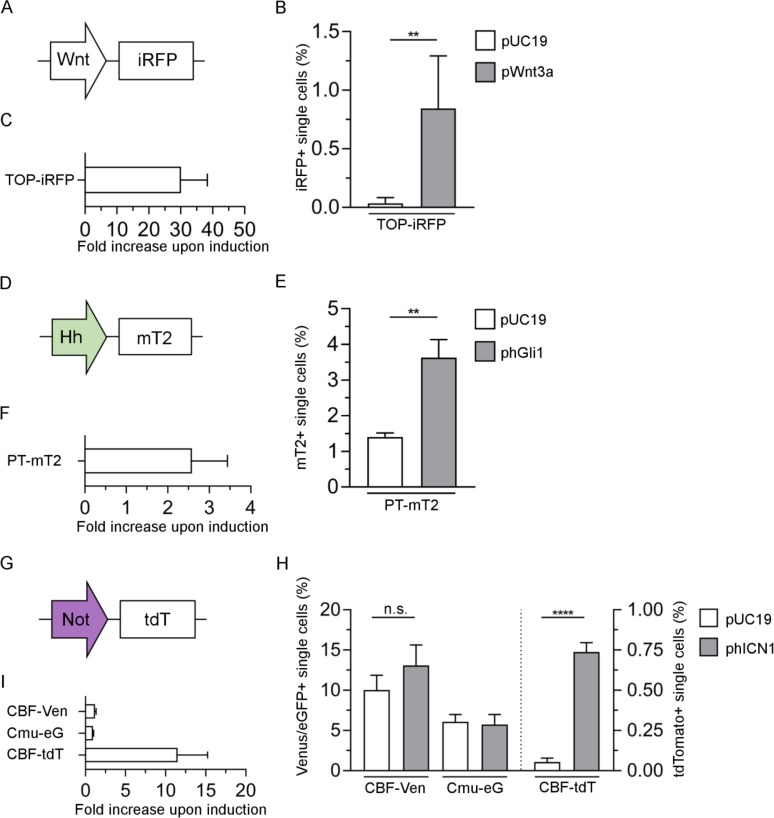
Evaluation of Wnt, Hh and Notch fluorescent single reporters via flow cytometry. (A) The Wnt promoter TOP was combined with iRFP. (D) The Hh sensitive promoter PTCH1 (wildtype promoter for the hedgehog receptor PTCH1 [30]) was joined with mTurquoise2 (mT2). (G) For the Notch (Not) reporter construct, the CBF promoter was combined with tdTomato (tdT). Transfection were carried out either in 293T cells (B, C, E, F) or HeLa (H, I). B, E, H: Fluorescence measurement of cells transfected with the indicated plasmid and co-transfected with either pUC19 control (white bars) or the indicated inducer plasmid (grey bars). C, F, I: fold increase in signal for the indicated plasmid upon co-transfection with pUC19 control versus inducer plasmid (C: pWnt3a; F: phGli1; I: phICN1). In B, E and H results are shown from a representative experiment; C, F and I are the average of 3–6 independent experiments. (**P≤0.01, ****P≤0.0001, ns = not significant, t-test, n≥4).

In a next step, fluorophore reporter genes were generated to evaluate specificity and signal dynamics. With the Wnt-construct TOP-iRFP (Fig 2A), baseline iRFP signal in 293T cells resulted in <0.04% iRFP^+^ cells, which increased significantly to a mean of 0.85% positive cells upon induction with pWnt3a (Fig 2B). This corresponds to a signal increase of around 30-fold (Fig 2C).

For the Hh reporter construct, the promoter PT wt was chosen (Fig 2D). The fluorophore reporter construct PT-mT2 resulted in approx. 3.5% mTurquoise2^+^ cells upon induction with phGli1, corresponding to an increase of 2.6-fold when compared to control (pUC19) transfected cells (Fig 2E and 2F). In addition, original (PT wt) and mutant binding versions of the promoter (PT mut, binding site BS2 replaced with linker sequence, see ref [30]) driving FLuc were compared (S3 Fig). Also here, signal increase was significantly higher when compared to mutant control.

As a Notch reporter protein, tdTomato was used and evaluated in HeLa cells (Fig 2G and 2I), as 293T cells did not show any signal increase upon induction with CBF-fluorophore construct (S4 Fig). In case of HeLa cells, CBF-tdTomato showed a highly significant, 11.5-fold signal increase upon induction. This was in contrast to the original plasmid reporter CBF-Ven, which in our hands only achieved a non-significant increase in the number of positive cells (1.2-fold, compared to 0.9 fold using the mutant binding site plasmid version Cmu-eG). Of note, CBF-tdT exhibited a baseline signal several times lower than that of both the original and mutant plasmid reporter versions CBF-Ven and Cmu-eG (Fig 2H).

Taken together, all fluorophore-reporter constructs showed significant signal increases upon induction, and satisfactory fold increases.

### Development of multicistronic pathway reporters

**Fig 3:**
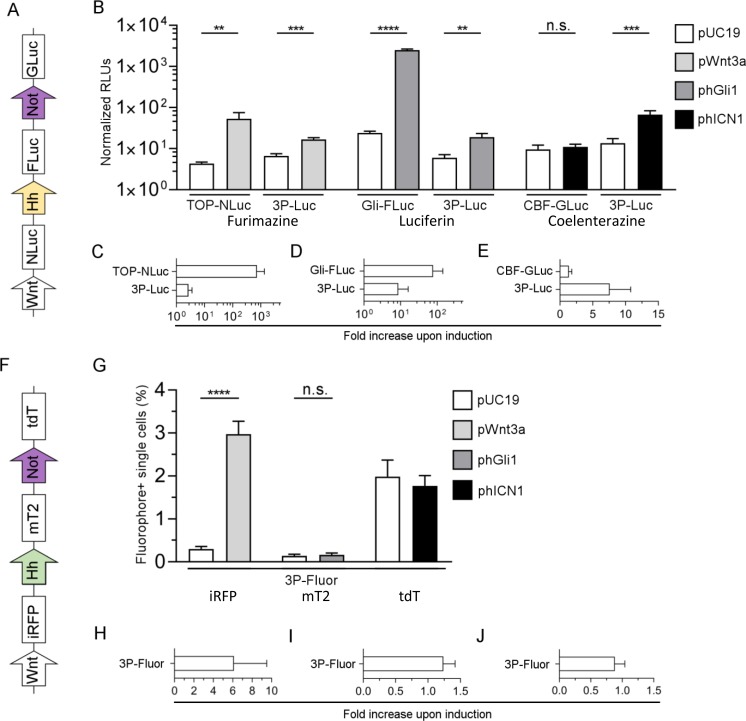
Evaluation of triple pathway reporter constructs. (A) The three single luciferase reporter gene plasmids for the Wnt, Hh and Notch pathways were joined by a gateway approach to yield 3P-Luc. (F) 3P-Fluor was generated from the three pathway reporter plasmids with indicated fluorophore reporters. Experiments in B, C, D, E, H and I were carried out in 293T cells. In panel G, 293T cell were used for pWnt3a and phGli1 and corresponding pUC19 control transfections, Hela were used in panel G for phICN1 and corresponding pUC19 control and in panel J. (B): Luminescence measurement of cells transfected with the indicated plasmid and co-transfected with either pUC19 control or the indicated inducer plasmid. All luciferase signals (B-E) were normalized on cell viability using the CellTiter Fluor® assay. Normalised RLUs were multiplied with 0.1 (Wnt-pathway experiments), 1 (Hh-pathway experiments) or 0.01 (Notch-pathway experiments). C-E: Quantification of fold increases for Wnt (C), Hh (D) and Notch (E). (G) Evaluation of compensated 3P-Fluor with a compensation matrix applied. Cells were transfected with 3P-Fluor and co-transfected with either pUC19 control or the indicated inducer plasmid. H-J: Quantification of fold increases of fluorescence positive cells for Wnt (H), Hh (I) and Notch (J). In B and G results are shown from a representative experiment; C, D, E, H, I and J are the average of 3–4 independent experiments. (**P≤0.01, ***P≤0.001, ****P≤0.0001, n.s. = not significant, t-test, n≥3).

Based on the modified Gateway cloning system described by Albers and colleagues [20], polycistronic plasmid vectors were designed and cloned utilising the Multiple Lentiviral Expression System Kit. The luciferase reporter 3P-Luc contained Wnt-NLuc, Hh-FLuc (using GLI-FLuc), and Notch-Gluc (using CBF-Gluc) expression cassettes, and in addition a PGK promoter driven Neomycin resistance for selection purposes and a CMV driven EGFP expression cassette for future sorting (for map see S1B Fig, scheme see Fig 3A). The yield of the resulting large plasmid (15,489 bp) was in the range of 4.8 μg per ml culture volume. In a separate set of experiments with single reporter plasmids we first ensured that there is no cross-reactivity between substrates, as it is known that NLuc can in principle also use colenterazine as a substrate catalysing the reaction to coelenteramide, and emit photons [45] (S5 Fig). In doing so, we transfected 293T cells either with TOP-NLuc or CBF-GLuc and analyzed the lysate of transfected cells with furimazine as substrate. Only in TOP-NLuc transfected cells a signal was detected, whereas in CBF-GLuc transfected ones no signal above background was measured. This indicated that GLuc cannot utilise furimazine as a substrate. When analysing lysates of similarly transfected cells, but with coelenterazine as a substrate, both TOP-NLuc or CBF-GLuc transfected cells gave a positive signal. This indicates that also NLuc can successfully use coelenterazine as a substrate. In the third control experiment, the supernatant of TOP-NLuc or CBF-GLuc transfected cells was analysed using coelenterazine as substrate. Here, only the supernatant from CBF-GLuc transfected cells gave a positive signal. Similar observations were made when co-transfecting cells with the respective inducer plasmid (i.e. TOP-NLuc plus pWnt3a or CBF-GLuc plus phICN1, data not shown). This altogether indicates that no or negligible NLuc is secreted, which could in principle also use coelenterazine as a substrate. The single luciferase reporter plasmids were then compared side-by-side with 3P-Luc (Fig 3B–3E). For all three pathways, induction was significant using the 3P-Luc plasmid. For the Wnt (Fig 3C) and Hh pathways (Fig 3D), signal induction was less pronounced when compared to the single reporters. Interestingly, Notch single reporter induction was not significant in these assays (Fig 3B).

The fluorescence reporter 3P-Fluor (Fig 3F) consisted of Wnt-iRFP, Hh-mT2 (using PT-mT2), and Notch-tdT. Using CMV-driven fluorophore variants for iRFP, mTurquoise2, tdTomato and eGFP, a compensation matrix was generated to minimise signal overspill between the individual colours. The matrices used showed a considerable interplay of mTurquoise2, eGFP and tdTomato (for 293T see S3 Table, for HeLa see S4 Table). Analyzing the compensated data, a robust increase of iRFP^+^ cells after induction of the Wnt pathway was observed, with a mean 6.1-fold increase (Fig 3G and 3H). In contrast to this, the data for the Hh (Fig 3G and 3I) and Notch pathway (Fig 3G and 3J) show neither a significant increase of fluorophore-positive cells upon induction, nor a fold-increase. Here, the detection of a clear-cut mTurquoise2^+^ or tdTomato^+^ subpopulation was not possible despite the application of compensation (S6 Fig).

### Small molecules evaluation

**Fig 4:**
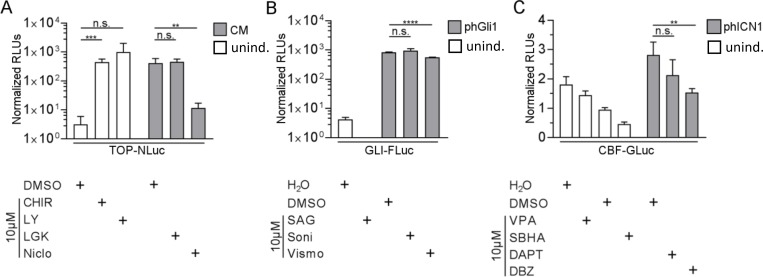
Small molecules evaluation with the novel reporters. 293T cells were transfected with the indicated reporter plasmid and treated with the listed compounds (10 μM). (A) TOP-NLuc; (B) GLI-FLuc, (C) CBF-Gluc. White bars: uninduced conditions (unind.), grey bars: induction with conditioned medium (A: CM, contains Wnt3a) or by co-transfection (B: phGli1, C: phICN1). CHIR = CHIR99021, LGK = LGK-974, LY = LY2090314, Niclo = niclosamide, Soni = sonidegib, Vismo = vismodegib. (**P≤0.01, ***P≤0.001, ****P≤0.0001, n.s. = not significant, t-test, n = 4) Normalised RLUs were multiplied with 100 (TOP-NLuc, GLI-FLuc and CBF-GLuc).

In an initial experimental series, several published small molecules were evaluated regarding their capability to induce or reduce the activity of single luciferase pathway reporters (Fig 4). 293T cells were transiently co-transfected with reporter and normalizing plasmid, and inducer plasmids where indicated. All compounds were tested at 10μM and added 4 hours after transfection. Activity was measured after 24 (Wnt) and 48 h (Hh, Notch), respectively. While CHIR99021 showed a significant TOP-NLuc induction, the activation seen with LY2090314 was not significant (Fig 4A). Niclosamide significantly decreased TOP-NLuc activity, while LGK-974 did not change the signal compared to solvent control.

After transfection with the Hh reporter GLI-FLuc, SAG did not induce the Hh pathway, and in fact gave lower FLuc signals than the solvent control (Fig 4B). In cells co-transfected with phGli1, only vismodegib significantly decreased Hh signalling.

Similarly, when tested on CBF-GLuc transfected cells, both of the two published Notch-activating substances, VPA and SBHA, rather decreased Notch signalling (Fig 4C). Of the two Notch inhibitors, only DBZ resulted in significant decrease of the GLuc signal in cells co-transfected with the activator phICN1.

### Evaluation of cross activation between pathways

**Fig 5:**
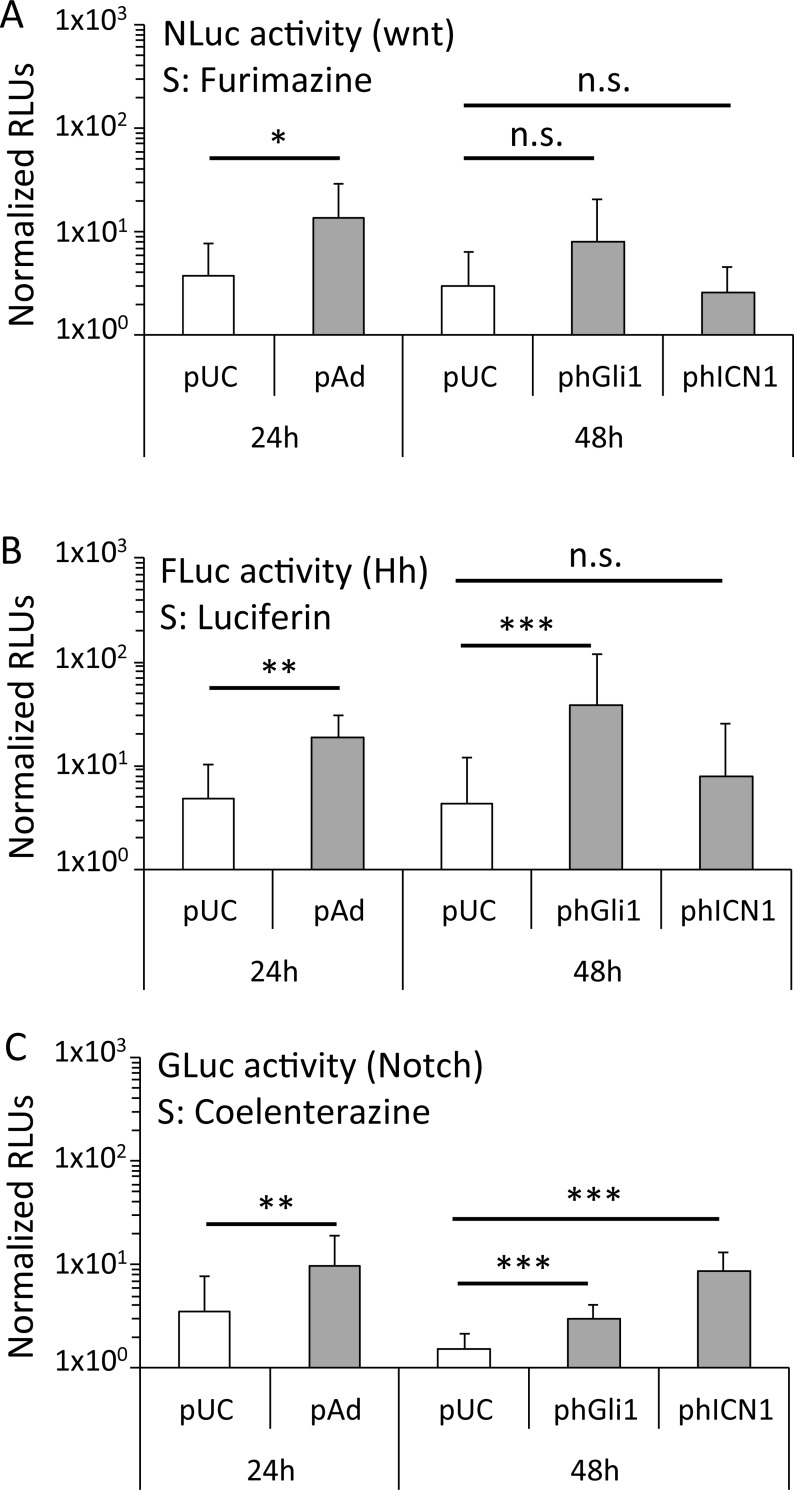
Cross-activation between Wnt, Notch and Hh studies with 3P-Luc. 293T cells were co-transfected with 3P-Luc and control plasmid pUC19 or the indicated inducer plasmid. Luciferase activity was determined 24h or 48h after transfection. (A) NLuc activity measured with substrate (S) furimazine in the lysate of cells (Wnt activity); (B) FLuc activity measured with substrate luciferin in the lysate of cells (Hh activity); (C) GLuc activity measured with substrate coelenterazine in the supernatant of cells (notch activity); *P≤0.05, **P≤0.01, ***P≤0.001, n.s. = not significant, two sided t-test, average from ≥ 2 independent experiments, n ≥ 6).

To study potential cross-induction effects between pathways, we quantified the activity of all three luciferases after induction of single pathways by co-transfection with 3P-Luc and the respective inducer plasmid (Fig 5).

293T cells were co-transfected with 3P-Luc and the indicated inducer plasmid (pWnt3a for wnt, phGli1 for Hh, phICN1 for Notch, pUC19 as control) and the signal was measured either 24h (pWnt3a co-transfection) or 48h (phGli1 and phICN1 co-transfection, respectively) thereafter. The activity of all three luciferases was determined for each induction, i.e. NLuc and FLuc in the lysate of cells, and GLuc in the supernatant. This ensured that there was negligible signal overspill between the luciferases. The induction of both Hh or Notch did not significantly activate Wnt (Fig 5A). In contrast, Hh activity was significantly increased when co-transfecting cells with the wnt inducer pWnt3a (Fig 5B), although it was unaffected by the Notch inducer phICN1. Finally, both, the Wnt inducer pWnt3a and the Hh inducer phGli1 also caused activity of Notch (Fig 5C).

## Discussion

In this work, we generated novel reporters for the Wnt, Hh and Notch pathway, with the overarching goal of creating triple pathway reporter constructs. Such reporters should be desirable for drug screening approaches, but also for investigating biological or pathological entities in real time. We have utilised mostly synthetic promoter elements, consisting of a repeat of identical cis-acting elements interspaced with stuffer sequences and placed either up- or downstream of a basic promoter. The TOPFlash promoter contains seven TCF/LEF binding sites next to a minimal TK promoter [27], 12GLI-RETKO-luc twelve Gli consensus sites in front of a minimal TK promoter [22] and CBF four CBF1 binding sites upstream of the basal simian virus-SV40-promoter [23]. Albeit such synthetic promoters allow high levels of transgene expression, this comes with the risk of increased background expression [46]. Hence, we ensured specificity by validating the constructs on 293T or HeLa cells. Induction of these respective pathways was accomplished by co-transfection with plasmids encoding for pathway activators. This method allows unambiguous proof of specificity due to the robust activation, cost effectiveness even for larger future screens (when compared to working with purified and commercially available proteins), and easy integration into our transfection workflow. Three different luciferases were chosen to be driven by Wnt, Hh and Notch pathway sensitive promoters, theoretically allowing signal analysis in parallel. All three inducer plasmids have been described in the literature to induce specific pathway activation by expression of the relevant protein. Plasmid pWnt3a was used in an adenoviral vector and induced expression of alkaline phosphatase in mesenchymal cells [28]. Plasmid phGli1 was used in a similar form by to overexpress Gli1 in in PANC1 cells, and the protein expressed measured by western blot [47]. Plasmid phICN is based on EF.hICN1.CMV.GFP [25]. Cells transfected with this plasmid overexpressed NICD1 protein [48]. Regarding the performance of our single reporters, we also investigated if sensitivity of the reporters is influenced by the type of luciferase used: Comparing TOPFlash (FLuc) and TOP-NLuc, induction levels were similar (Fig 1C), indicating that functionality of the constructs remains comparable. Performance between pathway reporters, however, varied: whereas Wnt-NLuc and Hh-FLuc were in the range of two log units, Notch-GLuc was only about 3-fold increased. In the literature, a promoter with four CBF binding sites in front of a full SV40 promoter driving FLuc was observed to have a 20–40 fold induction [11]. Nevertheless, we employed the current version with four CBF sites in front of a SV40 minimal promoter, which should result in lower background activity. Adding further binding sites could also improve the performance of the promoter, but, as mentioned above for Gli, this could also increase background activity [46].

We assumed that due to the signal amplifying effects of luciferase enzymes, the signal increase would be considerably reduced when pairing the same promoters with fluorophores. Bauer and colleagues directly compared TOP-GFP and TOP-Luc adding Wnt3a protein, estimating a >100-fold increase in both cases, although apparently saturating amounts of protein were added [49]. In another approach, a bidirectional NF-κB-responsive reporter was cloned simultaneously driving expression of FLuc and EGFP [50]. Upon stimulation with TNFα, luciferase activity increased several hundred fold, whereas the number of EGFP positive cells increase from 0.3 to 35% (although not considering the absolute EGFP expression per cell). From this, one could consider that luciferase based vectors offer a higher dynamic range, but fluorophore-based vectors are not necessarily less sensitive in terms of induction. In our experiments, the fold increase upon induction using fluorescent proteins decreased only to around one third (TOP and CBF promoter) and half (PT promoter), respectively. Still, it should be kept in mind that the half-life of reporter proteins largely differs, e.g. with 3h for cytoplasmic firefly luciferase [51] and >24h for EGFP [52]. To improve the dynamics of the readout, destabilised protein versions of the reporter proteins can be utilised [51, 52], although this could come with an overall decreased sensitivity. We also observed a dependence on the type of fluorophore used: although the original version CBF:H2B-Venus did not show significant signal increase upon induction, our CBF-tdTomato construct performed at considerably lower baseline with >10-fold induction (Fig 2I), which is even higher than the induction level obtained with the luciferase version CBF-GLuc (Fig 1I). Also, the cell type could apparently also influence the level of induction (i.e. weak Notch induction in 293T, 11-fold induction in HeLa using tdTomato as reporter).

In a next step, we generated triple pathway reporter plasmids with our constructs, which were then also evaluated. We used the approach described by Albers et al., based on Gateway cloning [20]. As backbone for the Gateway cloning approach, a lentiviral transfer plasmid was chosen, offering the possibility for transient transfection, but also the generation of stably transduced cell lines using a lentivirus. An additional CMV-EGFP cassette was added to enable sorting of transfected or transduced cells with FACS, as well as a neomycin cassette, adding an additional choice for cell selection (S1B Fig). For 3P-Luc, all luciferase activities had to be normalised to total cell viability, as normalization with CMV-driven luciferases was not possible, due to the genes already used as pathway reporters. The single reporters were included in these experiments, also to investigate differences due to the normalization method. For both single and triple reporter, the same normalization method was used in these experiments. With single reporters, we saw that the choice of normalization method (CMV-driven luciferases in Fig 1, CellTiter Fluor in Fig 3B–3E) had significant influence on the calculated induction level. TOP-NLuc showed >100-fold induction in Fig 1C and >700-fold induction in Fig 3C. With GLI-FLuc, the difference was less pronounced (200-fold in Fig 1F vs 70-fold in Fig 3D). The reaction of the single Notch reporter is remarkable, as it failed to reach a significant increase upon induction in Fig 3B, but had a highly significant response in Fig 1H, where we used another normalization approach. We thus assume that the alternative normalization method based on total cell viability instead of our usual co-transfection with CMV-driven luciferases, had an appreciable impact on the results. Investigation of the most appropriate normalization method would in itself be a separate study. Of note, we used equal total amounts of plasmid per well. Although this results in an up to 4.4-fold difference in terms of molar amounts of plasmid (e.g. 3P-Luc: 15.5 kb, CBF-GLuc: 3.5 kb), it makes sure that there is no difference in the needed amount of transfection reagent, thereby ensuring similar cellular viability and comparable results. Also, it is rather the total amount of nucleic acid complexed with the transfection reagent, e.g. polyethylenimine, which influences the transfection efficiency, and up to 90% of plasmid can be replaced by stuffer DNA still giving similar reporter gene expression levels [53]. Side-by-side comparison of induction levels between single reporter and 3P-Luc revealed remarkably lower levels for Wnt and Hh in 3P-Luc (700- vs 2.7-fold for Wnt, 70- vs 9-fold for Hh), albeit the same normalization method was used (CellTiter Fluor). Surprisingly, the Notch reporter performed better in the 3P-Luc construct (8-fold). The differences between single- and multiple pathway reporter are intriguing and can be explained in several ways. For example, all promoters, also our synthetic constructs, require the binding of transcriptions factors (TF) for activation. At least two effects can explain reduced activity of promoter elements within multicistronic vectors. Firstly, promoters interacting with similar TF will compete for these proteins [54]. Hence, it appears reasonable that single plasmids in an episomal status would be less prone to TF limitation when compared to several promoters in close vicinity in one plasmid. Secondly, steric hindrance can be an obstacle for TF binding [55], which makes it necessary to include spacer elements within TF binding sites in synthetic promoter elements. Such an effect is also possible in our case, even when different TF bind to the distinct promoter elements. However, in light of the aforementioned explanation, the increased activation of the Notch reporter on the 3P-Luc plasmid still appears contradictory.

With the ambitious aim of enabling pathway activity research on the live single cell level, we generated a fluorophore based triple reporter named 3P-Fluor. While the Wnt reporter iRFP was clearly inducible (6-fold increase in number of positive cells, compared to 30-fold with the single reporter), we were unable to detect a clear population of mTurquoise2^+^ and tdTomato^+^ cells, at induced as well as at control conditions. The reduction in iRFP signal, compared to the single pathway reporter plasmid, can be explained as for 3P-Luc. We assume that the strong absolute signal intensity of eGFP made compensation of existing, but faint mTurquoise and tdTomato signal impracticable, as our compensation matrices show the large overspill of eGFP in these channels. In case of tdTomato, the fluorophore was excited at 488 nm with only approximately 25% efficiency. This and the overall lower promoter strength when compared to CMV caused potentially too much signal overspill. The same can be assumed for mTurquoise2: while the 405 nm diode laser excites the fluorophore with approx. 50% efficiency, it also excited eGFP with approx. 17% (all spectra information obtained from https://www.fpbase.org/spectra/). Using flow cytometers with better-suited excitation wavelengths and emission filters would be one option. Alternatively, the CMV-EGFP cassette can be either eliminated and selection of transfected/transduced cells be carried out using neomycin. As another approach, either a weaker, constitutive active promoter or a destabilised EGFP version, e.g. D2EGFP [52], could be employed. Regarding the Hh promoter used, the presented data indicates that the 12GLI promoter outperforms the PTCH1 promoter. In a follow-up construct, we aim to implement a new 12GLI-mTurquoise2 reporter.

We also evaluated several small molecules to demonstrate the suitability of our single pathway reporters for such an approach. Only substances with reported activities for up- or downregulation were tested on respective pathways indicators. CHIR99021 and LY2090314 both inhibit GSK3 [36] and hence induced the Wnt reporter TOP-NLuc, albeit LY2090314 only non-significantly due to presumably potential toxicity of the compound [56]. LGK-974, a porcupine inhibitor, blocks Wnt secretion [36]. In accordance with literature, no effect of this compound was observed as Wnt pathway activation was facilitated externally by direct Wnt3a expression. Niclosamide did decrease the Wnt signal, as it induces DVL2 downregulation and LRP6 degradation [37].

The SMO-activating compound SAG did not induce our Hh-reporter, but as it was used at lower concentrations and measured within shorter timeframes, our setup might need optimization [38]. Sonidegib is a SMO antagonist [39], and as such does not interfere with our Gli-based pathway induction, explaining why we see no decrease in Hh pathway activity. Interestingly, vismodegib has a similar mode of action, but also inhibits the expression of Gli1/2 [40], supporting the inhibitory effect we saw in our assay.

VPA is a histone deacetylase, leading to the activation of Notch1, which in turn induces Notch signalling via CBF1 [44]. We saw no Notch pathway activation in our setup, which can be due to the cell line used [57]. For a future screen, other suitable cell lines can be chosen, e.g. highly malignant tumor cell lines. SBHA, which operates in a similar fashion [41], also failed to induce Notch signalling. The Notch pathway inhibitor DAPT blocks γ-secretase activity [42], and did not inhibit the Notch pathway in our assay. As we transfected cells to produce the Notch intracellular domain directly, thus bypassing the γ-secretase step, our findings are in line with the literature. DBZ, one of the most potent Notch inhibitors, affects the intracellular domain [43], which is in accordance with the reduced activity of our Notch reporter.

We were also interested to determine the utility of 3P-luc for measuring potential cross-activation between the pathways. This interplay can be of key importance in anticancer therapy, and could help to develop appropriate novel treatment regimens (for a summary of Wnt, Notch, Hh interplay and drug cross-reactivity, see ref [58]). When expressing wnt3A (via pWnt3a), we could observe both activation of Hh (i.e. binding of GLI to GLI-BS) and Notch (i.e. activation of the CBF promoter). The Wnt/Hh interplay is known to occur during embryonic development, but also in cancers [59]. Nakamura and colleagues could demonstrate, that Wnt activation by wnt3a induces the expression of GLI1 [60]. Hence, we conclude that also in our setup, induction of Wnt by wnt3a leads to Gli1 expression, which in turn binds to the 12Gli-RETKO promoter, activating the expression of FLuc. In a related way, Wnt can also interact with Notch signalling: CBF1 is a key transcription factor in Notch signalling, directly affecting the transcription of Notch related genes, and is activated after interaction with NICD. While the activation of the Notch receptor leads to proteolytic cleavage of its intracellular domain NICD, its intracellular actions and half-life is regulated by different posttranslational modifications, including phosphorylation and ubiquitinylation [61]. Its phosphorylation can also be catalysed by GSK3, although this leads to a reciprocal regulation of Wnt (due to concomitant beta-catenin phosphorylation and following degradation [62]). Still, there is also a direct interaction described between beta-catenin and NICD, which occurs also after Wnt activation with wnt3a [63]. Beta-catenin then increases the NICD activity by preventing its ubiquitinylation. Several studies demonstrated a close interaction between the hedgehog and the notch pathway [61]. Nevertheless, we could not find any study on the direct activation/upregulation of CBF-1 by Gli1. As our results clearly show activation of a CBF regulated promoter by Gli1 overexpression, such a connection can be expected and should be further studied.

For further studies, we have generated VSV-G pseudotyped lentiviral vectors with 3P-Luc and 3P-Fluor constructs as transfer plasmids. Cells stably transduced with these multi-reporter lentiviral vectors will be utilized for broader screening approaches.

Taken together, we report successful generation and evaluation of a multi-gene, luciferase-based three pathway reporter allowing the analysis of pathway specific regulation and cross-induction studies. Additionally, a functioning fluorescence based 3P-Fluor construct has the potential to enable *in vitro* screening approaches as well as in vivo applications using preclinical imaging. The fluorescence based reporter will need further optimization, but offers possibilities for studying the interplay of Wnt, Hh and Notch pathway and their response to treatment approaches on a single cell level. In principle, also 3P-Luc could be used for such microscopy based approaches, e.g. bioluminescence microscopy [64], although this would be limited to the non-secreted luciferases FLuc and NLuc.

## Supporting information

S1 Fig **Plasmid maps** of (A) CBF-GLuc and (B) 3P-Luc.
(TIFF)

S2 Fig Plasmid-based pathway inducer evaluation. 293T cells were transfected with (A) TOP-NLuc (substrate: furimazine) or (B) GLI-RETKO (substrate: luciferin); HeLa cells were transfected with (C) CBF-GLuc (substrate: coelenterazine). Co-transfections were carried out with the indicated inducer plasmids or pUC19/pDest (= pMuLE_Lenti_Dest_Neo) as control. (**P≤0.01, ****P≤0.0001, n.s. = not significant, t-test, n≥4). Normalised RLUs were multiplied with 100 (TOP-NLuc), 10 (GLI-RET) or 1000 (CBF-GLuc).
(TIFF)

S3 Fig Evaluation of PT wt and PT mut performance. (A) Representative experiment showing results of a co-transfection of the two plasmids upon induction with the Hh pathway activating plasmid phGli1 or the control plasmid pUC19 (PT wt: wildtype hPtch1 promoter; PT mut: hPtch1 promoter with inactive binding site for Gli). Normalised RLUs were multiplied with 100. (B) Quantification of signal fold increase upon induction over four independent experiments. (**P≤0.01, n.s. = not significant, t-test, n = 6).
(TIFF)

S4 Fig CBF-tdTomato performance in 293T cells. Upon induction of the Notch pathway via co-transfection with phICN1, no increase in tdTomato^+^ cells could be observed after 48h (n = 6).
(TIFF)

S5 Fig Cross reactivity study for substrates between NLuc and GLuc. 293 T cells were transfected either with TOP-NLuc (bright grey bars) or CBF-GLuc (dark grey bars) for 24h. Thereafter, supernatant was completely removed, 20 μL of supernatant incubated with coelenterazine assay reagent and bioluminescence measured (supernatant). Remaining cells were incubated with CellTiter Fluor reagent as described in materials and methods to determine total cell viability. After removing the CellTiter Fluor solution, remaining cells were lysed with 1x passive lysis buffer and bioluminescence measured using either furimazine (lysate) or coelenterazine (lysate). All signals are normalized for cell viability; mean values of two independent experiments are shown (n ≥ 6).
(TIF)

S6 Fig Compensated 3P-Fluor has no discernible positive population for mTurquoise2 or tdTomato. (A) 293 T cells were co-transfected with PT-mT2 and phGli1, and an mTurquoise2^+^ population can be easily detected. In contrast, (B) the gating approach of 3P-Fluor and hGli1 co-transfected cells show no clear-cut population, and gating was tentative. Similarly, (C) a small and reproducible tdTomato^+^ population was discernible in CBF-tdTomato and EF.hICN1 co-transfected HeLa cells, but (D) not present in compensated 3P-Fluor and EF.hICN1 co-transfected samples. Representative samples shown.
(TIFF)

S1 File Sequence file for CBF-GLuc. (GB)

S2 File Sequence file for 3P-Luc. (GB)

S1 Table Addgene information of generated plasmids. (XLSX)

S2 Table Names of plasmids used for different pathways. (XLSX)

S3 Table Compensation matrix used for 293T cells. (XLSX)

S4 Table Compensation matrix used for HeLa cells. (XLSX)
