# Supplementary figures and images for: Luminescent and fluorescent triple reporter plasmid constructs for Wnt, Hedgehog and Notch pathway
Source: PLoS One. 2019 Dec 20;14(12):e0226570. doi: 10.1371/journal.pone.0226570 (PMC6924688; doi:10.1371/journal.pone.0226570)

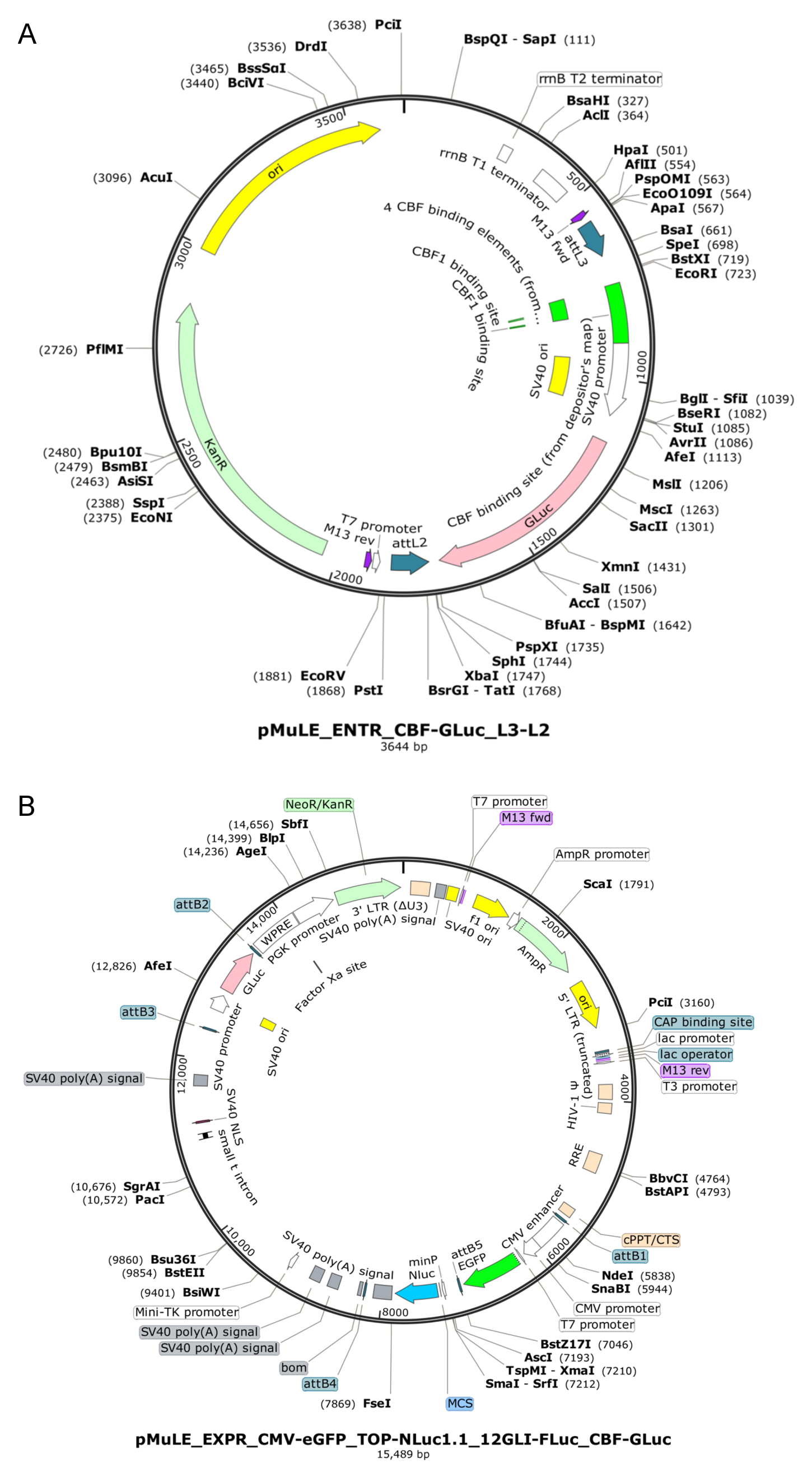

Supplement: S1 Fig — Plasmid maps of (A) CBF-GLuc and (B) 3P-Luc. (TIFF) [file pone.0226570.s001.tiff]

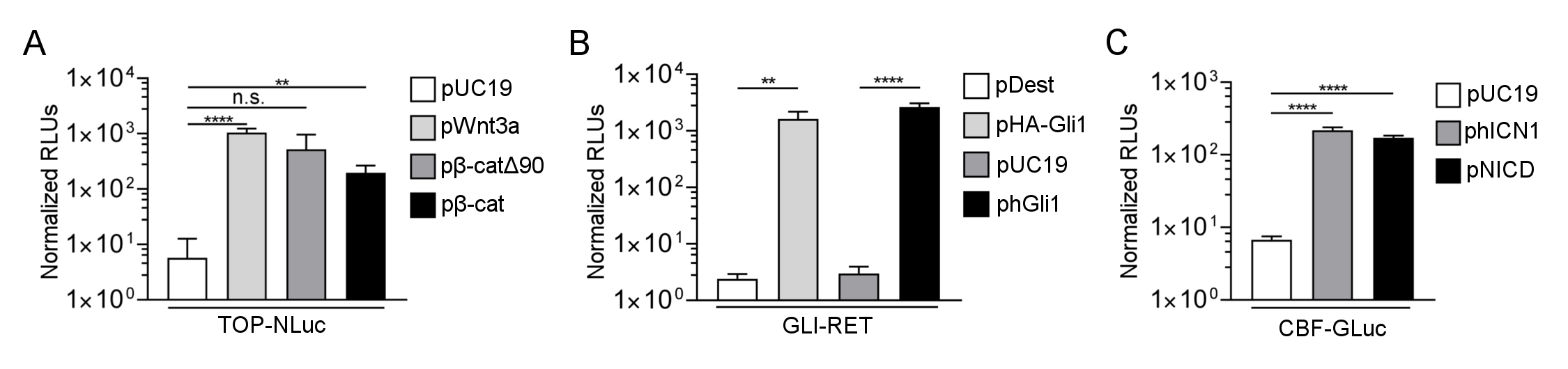

Supplement: S2 Fig — 293T cells were transfected with (A) TOP-NLuc (substrate: furimazine) or (B) GLI-RETKO (substrate: luciferin); HeLa cells were transfected with (C) CBF-GLuc (substrate: coelenterazine). Co-transfections were carried out with the indicated inducer plasmids or pUC19/pDest (= pMuLE_Lenti_Dest_Neo) as control. (**P≤0.01, ****P≤0.0001, n.s. = not significant, t-test, n≥4). Normalised RLUs were multiplied with 100 (TOP-NLuc), 10 (GLI-RET) or 1000 (CBF-GLuc). (TIFF) [file pone.0226570.s002.tiff]

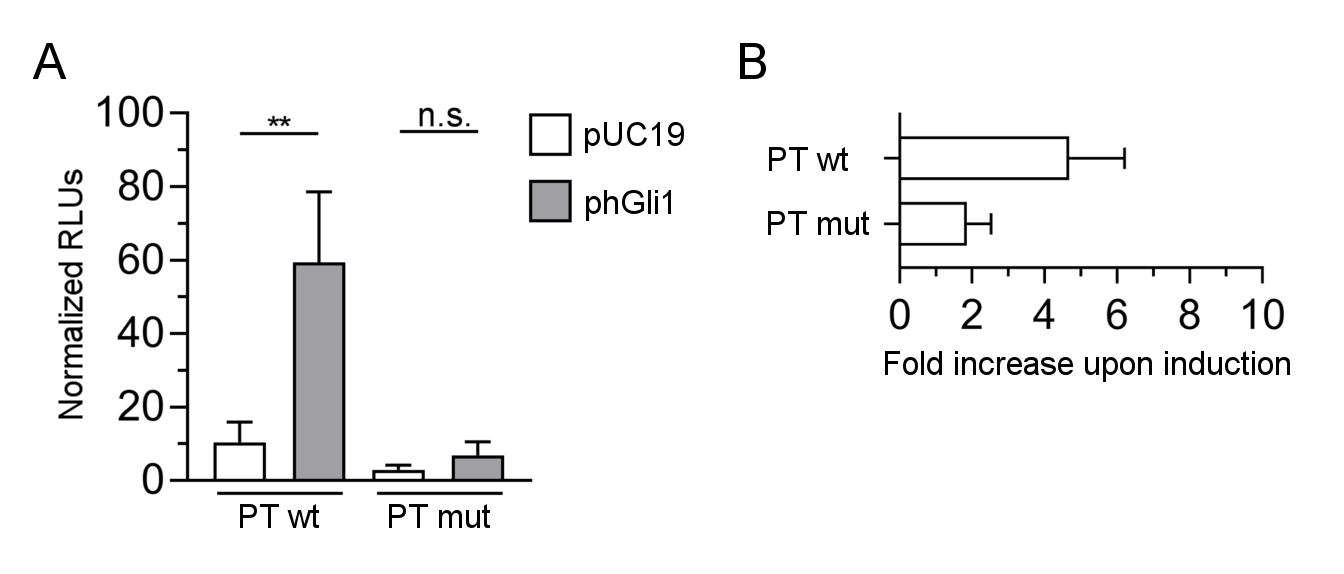

Supplement: S3 Fig — (A) Representative experiment showing results of a co-transfection of the two plasmids upon induction with the Hh pathway activating plasmid phGli1 or the control plasmid pUC19 (PT wt: wildtype hPtch1 promoter; PT mut: hPtch1 promoter with inactive binding site for Gli). Normalised RLUs were multiplied with 100. (B) Quantification of signal fold increase upon induction over four independent experiments. (**P≤0.01, n.s. = not significant, t-test, n = 6). (TIFF) [file pone.0226570.s003.tiff]

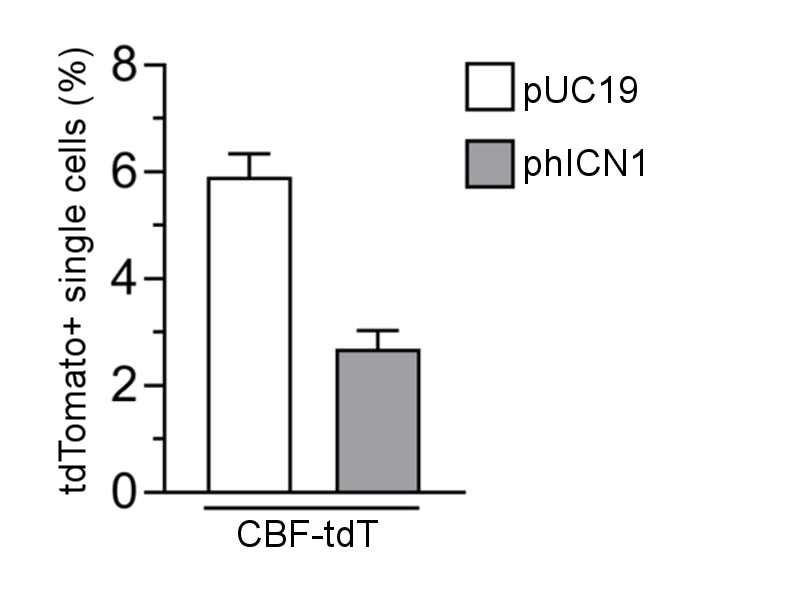

Supplement: S4 Fig — Upon induction of the Notch pathway via co-transfection with phICN1, no increase in tdTomato+ cells could be observed after 48h (n = 6). (TIFF) [file pone.0226570.s004.tiff]

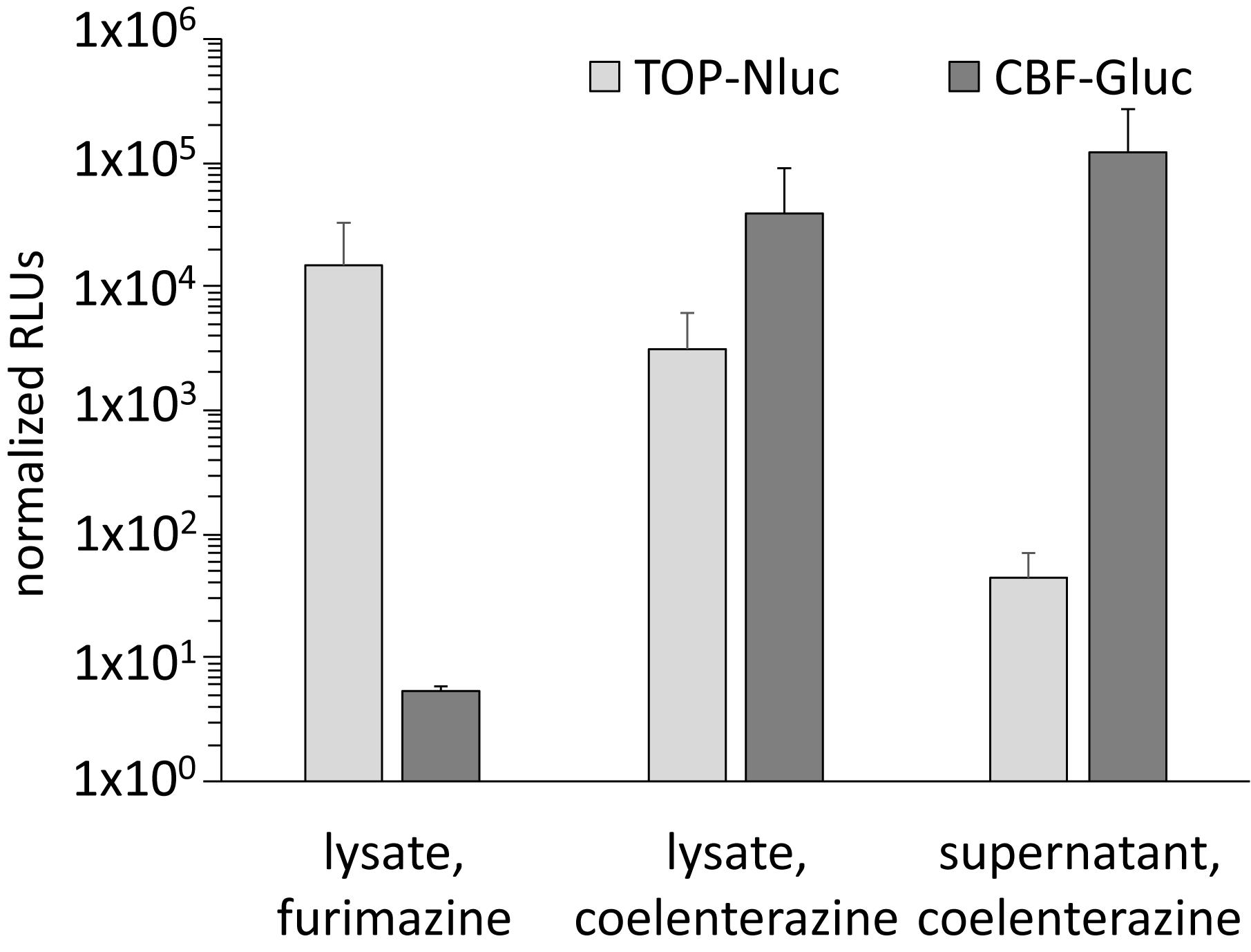

Supplement: S5 Fig — 293 T cells were transfected either with TOP-NLuc (bright grey bars) or CBF-GLuc (dark grey bars) for 24h. Thereafter, supernatant was completely removed, 20 μL of supernatant incubated with coelenterazine assay reagent and bioluminescence measured (supernatant). Remaining cells were incubated with CellTiter Fluor reagent as described in materials and methods to determine total cell viability. After removing the CellTiter Fluor solution, remaining cells were lysed with 1x passive lysis buffer and bioluminescence measured using either furimazine (lysate) or coelenterazine (lysate). All signals are normalized for cell viability; mean values of two independent experiments are shown (n ≥ 6). (TIF) [file pone.0226570.s005.tif]

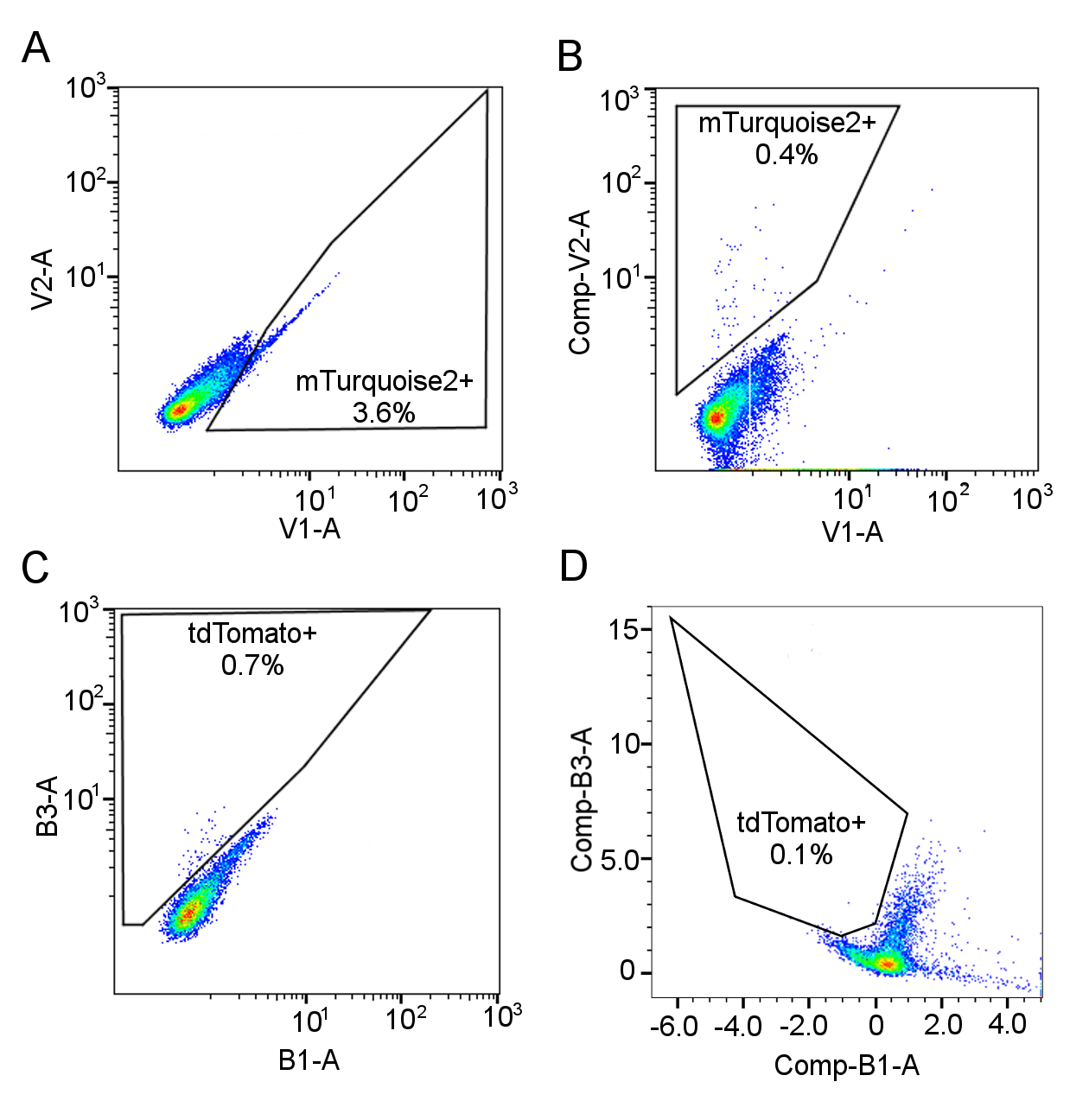

Supplement: S6 Fig — (A) 293 T cells were co-transfected with PT-mT2 and phGli1, and an mTurquoise2+ population can be easily detected. In contrast, (B) the gating approach of 3P-Fluor and hGli1 co-transfected cells show no clear-cut population, and gating was tentative. Similarly, (C) a small and reproducible tdTomato+ population was discernible in CBF-tdTomato and EF.hICN1 co-transfected HeLa cells, but (D) not present in compensated 3P-Fluor and EF.hICN1 co-transfected samples. Representative samples shown. (TIFF) [file pone.0226570.s006.tiff]
